# The Impact of Urbanization on the Distribution of Spontaneous Herbaceous Plants in an Ancient City: A Pilot Case Study in Jingzhou, China

**DOI:** 10.3390/plants12193353

**Published:** 2023-09-22

**Authors:** Shuwang Hou, Changwei Tian, Jianghui Meng, Chunyan Liu, Zhen Yao

**Affiliations:** College of Horticulture and Gardening, Yangtze University, Jingzhou 434025, China; houshuwang@163.com (S.H.); t75t4856956@163.com (C.T.); mengjianghui1239@163.com (J.M.); 201573031@yangtzeu.edu.cn (C.L.)

**Keywords:** spontaneous herbaceous plant, urban biodiversity, urban greening, urbanization, habitat, ancient city

## Abstract

Spontaneous herbaceous plants (SHPs) play an essential role in urban biodiversity. Research on the diversity of SHPs has profound implications for the conservation of urban biodiversity and green space management in the process of urbanization. We investigated the habitat, life form, and growth form of SHPs by combining samples and inspections in Jingzhou, in central southern China. Additionally, we chose three typical regions—Ji’nan, Gucheng, and Shashi—for the examination and comparison of biodiversity. The results showed that diverse habitats provided abundant living space for SHPs of different growth forms and life forms in Jingzhou. Water edges with higher humidity do not significantly support more SHP growth forms and life forms, except for pseudo-rosette, partial-rosette, and perennial plants. In addition, both wasteland and road gaps and slopes support significantly more SHP growth forms, including erect, tussock, and others. Wasteland supported the vast majority of species, both growth forms and life forms. In the diverse habitats, there are 352 plant species belonging to 70 families and 236 genera in Jingzhou (Ji’nan 184 species, Gucheng 157 species, and Shashi 127 species). Plant species diversity differed according to the level of management. The Ji’nan region had a large number of SHP species because of the less disruptive and milder management implemented in this region. SHPs show good performance and can provide wild landscape effects; therefore, they have the potential to be used in many urban landscaping applications. In the process of urbanization expansion, we should implement the concept of protection and coordinated development in new construction areas. Our study has important implications for the support of SHPs in urban areas.

## 1. Introduction

Urbanization is one of the main causes of global biodiversity loss [1]. The change in plant communities caused by land cover change and biological invasion is generally considered a negative consequence of urbanization. Fragmentation caused by land cover change is the main cause of biodiversity loss, mainly manifested as habitat loss and modification, which inhibit native species in urban areas [2,3]. Urbanization also enables the substantial migration of non-native plant species (most of which are cultivated plants) into urban areas, which is largely facilitated by human activities [4,5,6]. The arrival of non-native species encroaches on the living conditions of native species and can damage the original ecology. The process by which non-native species replace native species results in biological homogenization [7,8]. Balancing the relationship between urbanization and biodiversity conservation has become an important issue.

Spontaneous herbaceous plants (SHPs) are composed of herbaceous plants that are not intentionally planted by humans and may not be remnants of natural habitats [9]. They can not only enhance the naturalness of urban landscapes but also promote biodiversity [8]. In addition to improving the environment through the creation of carbon sinks, SHPs also play a vital role in the carriage of traditional culture [10]. Further, SHPs in urban areas can provide ecosystem services, support urban wildlife, and improve human health and well-being [11,12]. Studying changes in the species structure and characteristics of SHPs in the process of urbanization is instrumental in protecting plant diversity in environments under strong human interference [13].

Research into spontaneous vegetation (trees, shrubs, and herbs) in urban areas has focused on the relationship between urban green space properties and spontaneous species in green spaces, the development of spontaneous vegetation recognition algorithms, the driving factors of urban spontaneous vegetation, and the relationship between urban spontaneous vegetation and residents [14,15,16,17,18,19]. With the concept of ecological cities and urban wilderness being increasingly accepted, urban SHPs are receiving increased attention. For example, Zhang et al. (2023) [20] studied the impact of landscape structure in urban ecological corridors on the diversity of SHPs, and Ren et al. (2022) [21] showed that tree cover can improve species richness of SHPs underneath and have a large impact on the interspecific coexistence relationship in a built-up area. In previous studies, the diverse personality patterns and driving factors of SHPs have been explored at the urban or regional scale [14,22,23]. However, the impact of urbanization on the distribution of SHPs is still unclear. Moreover, there have been few studies linking the distribution of growth and life forms of SHPs supported by different habitats.

In recent years, many countries and regions have studied vegetation diversity in local heritage cities [24,25,26]. Urban biodiversity studies have been conducted in many cities in China, mainly focusing on urban plant communities, urban greening management, habitat connectivity, and the drivers of spontaneous plant abundance [9,14,27,28]. Jingzhou is an ancient city in central southern China with a 2000-year history [29]. It has the only complete ancient city wall in southern China and has many sanctuaries for native plants. With increased urbanization and economic growth, Jingzhou’s development and reconstruction area is large and dispersed, with several temporary wastelands and undeveloped land. Additionally, because of the distinct land use history, different regions of Jingzhou have diverse land use patterns and management levels, which have various effects on the support for SHPs. Meanwhile, there is little information and research on the history of land use and the survival of SHPs in Jingzhou. Under these circumstances, SHPs face the risk of habitat loss and a reduction in species diversity. Jingzhou was selected as a representative area to explore the role of spontaneous herbaceous plants in the process of urbanization and their protection methods.

**Figure 1:**
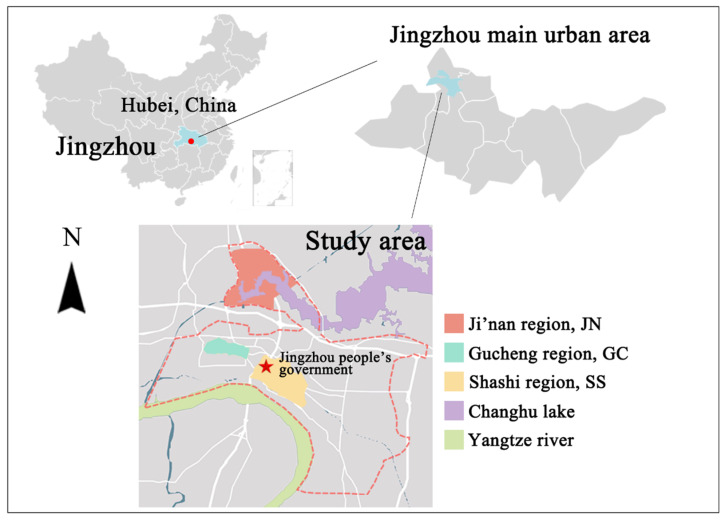
Study area location.

In the present study, we investigated the differences in growth forms and life forms of SHPs supported by the diverse habitats caused by urbanization construction. The composition and distribution of SHP communities were investigated in the spring and autumn by sampling and inspection (study area location is shown in Figure 1). In addition, we selected three typical regions under different management levels to investigate the distribution of SHP diversity. Through this study, we explored how to treat SHPs in ancient cities during the process of urbanization.

## 2. Results and Analysis

### 2.1. The Influence of Habitat on the Growth Form and Life Form of SHPs

**Figure 2:**
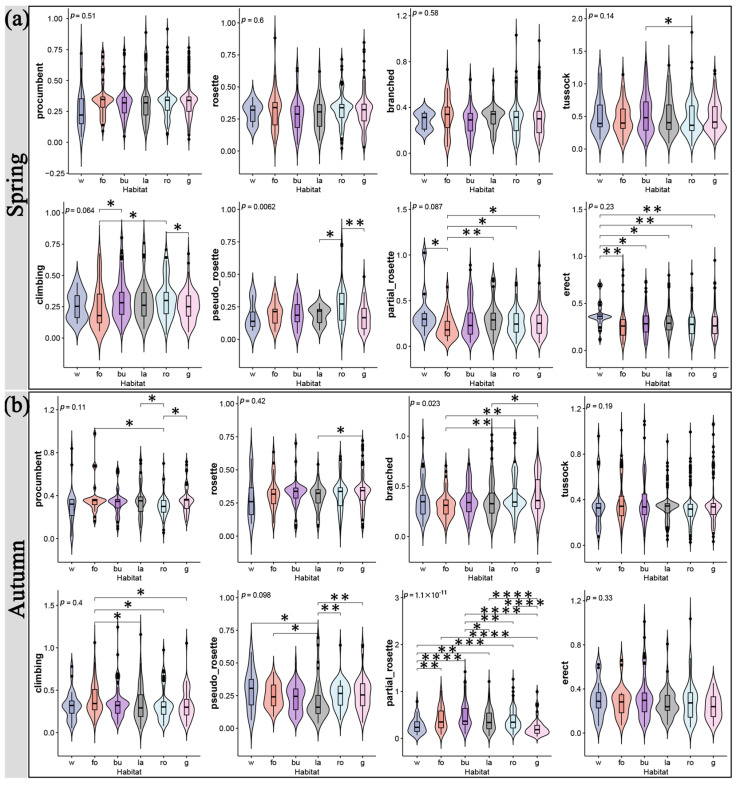
The influence of different habitats on the Shannon diversity index of distinct SHP growth forms in spring (**a**) and autumn (**b**). w, water edge; fo, forest edge and gap; bu, building waste and idle land; la, wasteland; ro, road gap and slope; g, garden. Asterisks indicate statistically significant differences between habitats as determined using the Wilcoxon rank sum test. *, *p* < 0.05; **, *p* < 0.01; ***, *p* < 0.001; ****, *p* < 0.0001.

**Figure 3:**
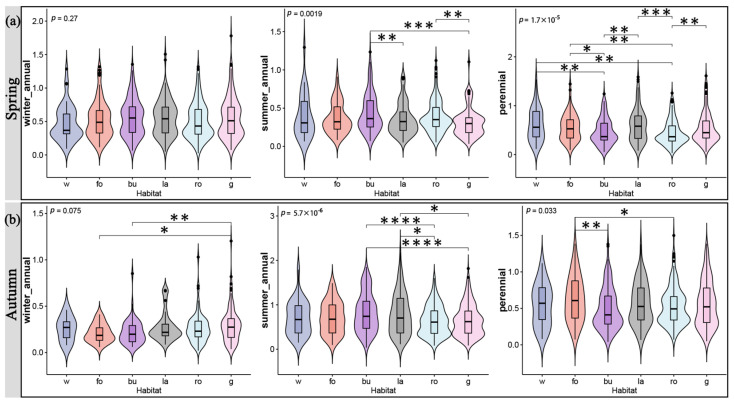
The influence of different habitats on the Shannon diversity index of distinct SHP life forms in spring (**a**) and autumn (**b**). w, water edge; fo, forest edge and gap; bu, building waste and idle land; la, wasteland; ro, road gap and slope; g, garden. Asterisks indicate statistically significant differences between habitats as determined using the Wilcoxon rank sum test. *, *p* < 0.05; **, *p* < 0.01; ***, *p* < 0.001; ****, *p* < 0.0001.

**Figure 4:**
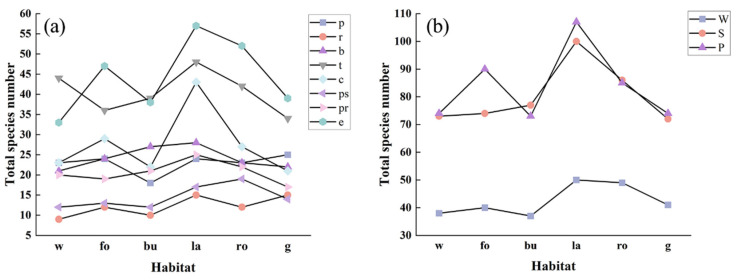
The total number of species supported by each habitat. (**a**) Growth form; (**b**) life form. Habitat: w, water edge; fo, forest edge and gap; bu, building waste and idle land; la, wasteland; ro, road gap and slope; g, garden. Growth form: p, procumbent; r, rosette; b, branched; t, tussock; c, climbing; ps, pseudo-rosette; pr, partial rosette; e, erect. Life form: W, winter annual; S, summer annual; P, perennial.

Different habitats had varying effects on various growth forms and life forms. The seasons also affected this difference. In terms of the growth form, in the spring, the forest edge and gap significantly supported more species with a partial rosette growth form, while the water edge supported fewer species with an erect growth form (Figure 2a). Autumn saw a substantial difference between the branched species supported by the forest edge and gap and those supported by the road gap and slope and garden, while the pseudo-rosette species supported by the wasteland outperformed those supported by the road gap and slope and garden (Figure 2b). However, across the six different types of habitats, partial-rosette species showed significant differences in the fall (Figure 2b). For the life form, there were significant differences in perennial species among the six types of habitats, with the summer annual species supported by building waste and idle land being much higher in number than those supported by wasteland and road gap and slope in spring (Figure 3a). The summer annual species supported by road gaps and slopes were substantially less numerous in autumn than those supported by building waste and idle land and wasteland (Figure 3b). In total, with the exception of procumbent, pseudo-rosette, and rosette species, wasteland generally supported the most SHP growth forms (Figure 4a). The majority of procumbent species were found in gardens, whereas the majority of pseudo-rosette species were found on road gaps and slopes (Figure 4a). Consistent with the growth form results, wasteland supported the most life forms of SHPs, while winter annual species had the fewest (Figure 4b).

### 2.2. Composition of SHPs in Jingzhou and in the Three Regions

**Figure 5:**
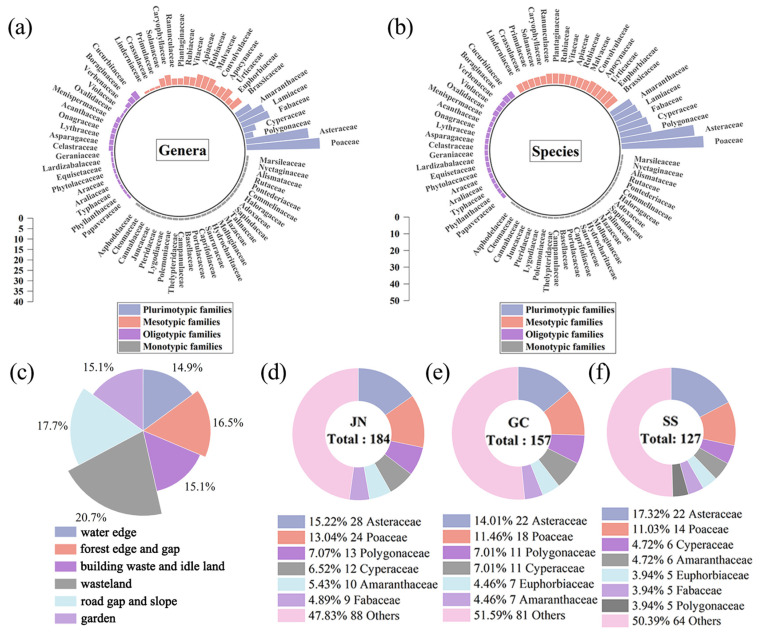
Composition and statistics of SHPs of Jingzhou. (**a**) Genera; (**b**) species; (**c**) habitats; (**d**) Ji’nan region; (**e**) Gucheng region; (**f**) Shashi region. Plurimotypic families, families with more than 10 species; mesotypic families, families with 5–9 species; oligotypic families, families with 2–4 species; monotypic families, families with only 1 species.

Habitats supported a large number of species in Jingzhou. A total of 352 SHPs from 70 families and 236 genera were recorded in Jingzhou. Families with ≥10 species were ranked by the number of species: Poaceae (35 genera, 49 species), Asteraceae (30 genera, 44 species), Polygonaceae (4 genera, 19 species), Cyperaceae (6 genera, 18 species), Fabaceae (14 genera, 16 species), Lamiaceae (13 genera, 15 species), and Amaranthaceae (8 genera, 14 species), which together comprised 175 species (49.7% of the total number of species) belonging to 110 genera (Figure 5a,b). A complete list of the 352 species records is presented in Supplementary Table S1. Among the habitats, wasteland supported the most species (20.7%) (Figure 5c).

In the records for the three regions, Ji’nan had the largest number of SHP species (184 species). The family components of the three regions are shown in Figure 5d–f. Plant species from the families Asteraceae and Poaceae in the three regions account for the largest share, accounting for 28.3%, 25.5%, and 28.3%, respectively, which is consistent with the overall findings for Jingzhou.

### 2.3. Composition of Growth Form, Life Form, and Species Diversity Distribution in Three Regions

**Figure 6:**
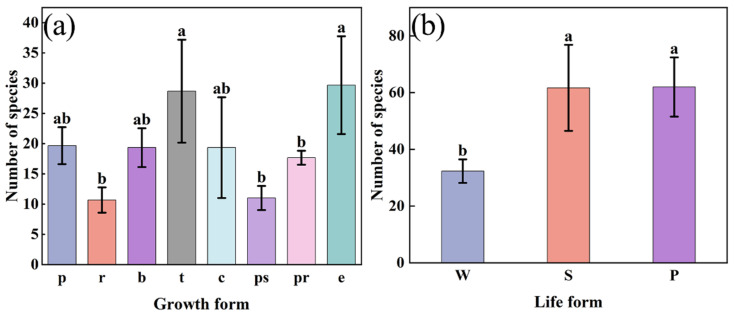
Species composition among growth forms (**a**) and life forms (**b**) of SHPs in three regions. p, procumbent; r, rosette; b, branched; t, tussock; c, climbing; ps, pseudo-rosette; pr, partial rosette; e, erect. W, winter annual; S, summer annual; P, perennial. Data are means ± SD of the three regions’ replicates. Different lowercase letters indicate statistically significant differences between regions as determined by ANOVA using the Tukey–Kramer post hoc test (*p* < 0.05).

Three growth types, tussock, climbing, and erect, exhibited large differences in the number of species in the three regions (Figure 6a). Among the small growth forms in the three regions, tussock and rosette are significantly different (*p* < 0.05). The value of erect is the largest among the large growth forms, which is significantly different from pseudo-rosette and partial rosette (*p* < 0.05). For the life forms of plants in the three regions, winter annual presented significant differences with summer annual and perennial (*p* < 0.05) (Figure 6b). Summer annual and perennial plant life forms dominated in all three regions, but they exhibited a higher proportion in Ji’nan and a lower proportion in Shashi (Figure 6b).

**Figure 7:**
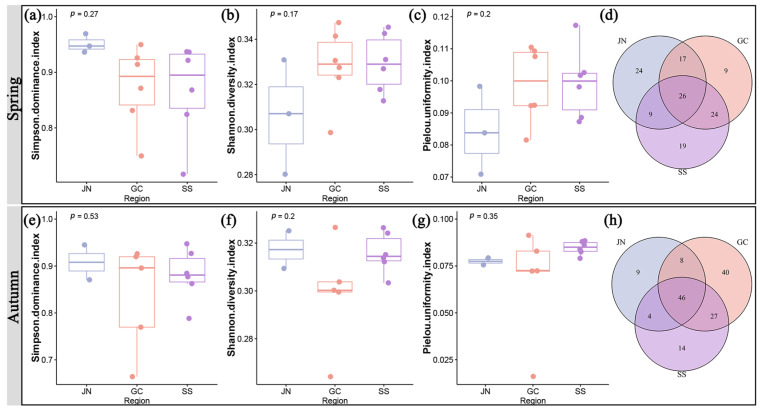
Difference in species diversity: Simpson dominance index (**a**,**e**), Shannon diversity index (**b**,**f**), Pielou uniformity index (**c**,**g**), and Jaccard index (**d**,**h**) in three regions in spring and autumn. JN, Ji’nan region; GC, Gucheng region; SS, Shashi region. In the box diagram, the top and bottom lines represent 1.5 IQR, the upper and lower lines of the box represent the upper quartile and lower quartile, and the line inside the box represents the median.

Although Ji’nan contained the most species, it did not exhibit significantly high diversity and evenness of SHPs. The Simpson dominance index of Ji’nan was higher than that of Gucheng and SS in both the spring and the autumn (Figure 7a,e). The Ji’nan region had a lower value than the Gucheng and Shashi regions in terms of the Shannon diversity index in the spring, but the difference between the three regions’ values was negligible in the autumn (Figure 7b,f). The Pielou uniformity index of the three regions displayed varying outcomes in the spring and autumn. The Pielou uniformity index of Ji’nan was low in the spring, but the three differences were less noticeable in the autumn (Figure 7c,g). This result may be due to the exchange of plant communities and propagation of propagules between the three regions. Regarding beta diversity, Ji’nan–Gucheng and Ji’nan–Shashi shared fewer species than Gucheng–Shashi did in the spring and autumn, demonstrating a higher tendency in Ji’nan’s species diversity, while the degree of species similarity between the Gucheng and Shashi regions was higher (Figure 7d,h).

## 3. Discussion

### 3.1. Diverse Habitats Provide Abundant Living Space for SHPs of Different Growth Forms and Life Forms

The diverse habitats in a city offer plants an opportunity to survive [30]. Our study provided a list of urban spontaneous herbaceous diversity for Jingzhou in south central China, and 352 plant species belonging to 70 families were recorded (Figure 5 and Figure S1). Jingzhou’s record considerably outstrips the statistics, surpassing the city with the highest number of documented species (284 species), and is more than the median number of SHPs reported in China, which is 116 [8]. This comparison demonstrates the abundance of SHP resources in the main urban area of Jingzhou.

Jingzhou, on the Yangtze River’s banks, has a variety of wetland habitats for plants. More roads, building areas, and wastelands arise as the urbanization process, ground hardening, and the influence of the local microclimate progress, and the living spaces of SHPs adapted to the wetland environment are constricted [3,14]. Our findings indicated that water edges with higher humidity do not significantly support more SHP growth forms and life forms, except for pseudo-rosette, partial-rosette, and perennial plants (Figure 2 and Figure 3). At the same time, urbanization and construction support another group of SHPs that can survive in dry, arid settings [2]. According to our results, both wasteland and road gap and slope supported significantly more SHP growth forms, including erect, tussock, and others, with wasteland supporting the vast majority of species (Figure 4). When comparing the life forms of species, the less supported winter annual plants can be linked to climate, while the difference between summer annual and perennial plants can also be attributed to regular green space management [31,32].

Urbanization and modernization provide the possibility of alien plant migration. The survival of native plants is severely affected by the presence of alien plants [33]. The niche of the plant community is incomplete, and there are many vacancies; therefore, it is easier for the area to be invaded by invasive plants [34,35]. Nearly 20% of the plants in the three regions in Jingzhou were alien invasive species, similar to the situation in other cities in China [14,36]. Plants with higher invasion levels, such as *Bidens pilosa* and *Solidago canadensis*, pose a threat to the survival of native species. We found that invasive species occupied a large area of habitat in each of the three regions, which should have belonged to the native species. Invasive plant origin is not related to the region of invasion [33], as the species of invasive plants in the three regions overlapped.

In line with the previous studies, additionally, we found that the Poaceae and Asteraceae have the greatest number of species at the city and regional scales [8,9]. Most species of the two families have a large ecological niche and status as the principal angiosperm families among herbaceous plants, which enables them to adapt to the diverse and barren habitats of urban areas and be extensively distributed [8,37].

With urbanization, some kinds of SHPs may vanish, and this decline may be irreversible [1]. Lei et al. [38] found 267 types of spontaneous angiosperms in a survey of Jingzhou ancient city wall vegetation (near Gucheng region), including rare plants such as *Spiranthes sinensis*. Comparing this list with our study, much of the native vegetation in Jingzhou no longer existed after more than 20 years of urbanization.

### 3.2. The Diversity of SHPs in Urban Areas Is Influenced by the Level of Management

Different levels of management affect the biodiversity of urban areas. Lerman et al. [39] demonstrated that different lawn mowing frequencies have an impact on the biomass of grass, which in turn affects the diversity of bees in lawns. In the present study, we selected three typical regions of Jingzhou with different management and land use histories and assessed species richness and diversity. The difference in the three regions’ alpha diversity was not significant, and we deduced that each region’s level of management is to blame for this result. As a mature urban system, Shashi has a high level of green space management and large human interference. Regular mowing and seasonal replacement of cultivated materials in the green spaces, as well as ongoing construction and cleaning of these spaces, have a huge impact on the growth of SHPs [40]. Human management plays a similar role as an environmental sieve for SHPs. Plants that remain in the urban system are usually resistant to drought, barrenness, and low light and have seasonal characteristics, short growth seasons, short life cycles, and hibernation [41]. As a cultural heritage and tourism area under development, Ji’nan is situated in close proximity to both suburban and rural regions with a shorter history of land urbanization construction but rather a history of farmland, free from significant human interference. Additionally, it boasts a rich water system, has relatively more native vegetation, and exhibits less damage compared to the other two regions [29]. This enables Ji’nan to support a greater number of species than Gucheng and Shashi. However, with development and expansion, plant survival in this area is at risk. Additionally, as a mature cultural tourism region, although the Gucheng has a complete ecological corridor landscape, the Jingzhou ancient city wall has high plant species uniformity due to human interference. The SHPs in the Gucheng tourism region are mowed regularly to achieve a neat ornamental effect, affecting the natural succession of the spontaneous herbaceous community.

Human management can also lead to species homogenization [2], which is confirmed by the Jaccard similarity index of Gucheng-Shashi. More species were shared between Gucheng and Shashi, and there was a greater degree of species similarity between the Gucheng and Shashi regions. This may be due to their high levels of human interference and similarly patchy or mosaic plant habitats [3]. It is worth noting that the Jaccard similarity index of Ji’nan was low, which means that the species supported by Ji’nan have differences from Gucheng and Shashi, supporting the particularity of habitat and the protection of SHPs in Ji’nan as compared to the two other regions from another perspective.

### 3.3. Protection and Management of SHPs in the Process of Rapid Urbanization

Rapid expansion of urbanization and urban renewal iterations have resulted in many problems, including a large amount of building waste, idle land, and abandoned factories [42,43]. During the process of abandonment or in idleness, the autophyte community naturally develops and gradually occupies a site. Subsequently, rich plant communities form a rewilding landscape in the original site, which can improve ecosystem resilience and maintain biodiversity [44,45]. The rational use of these plant communities to repair the damaged environment can provide a more ecological landscape for urban green spaces [46]. The study indicated that, in addition to the Poaceae and Asteraceae families, the dominant families also included Cyperaceae, Polygonaceae, and Fabaceae (Figure 5). For the restoration of urban ecology and the construction of unique rewilding urban landscapes, the families of Poaceae and Cyperaceae can be adopted for the beauty of wilderness [47]. In addition to ornamental value, Fabaceae and Polygonaceae species provide insect habitats and nectar sources, possessing ecological functions [11,48]. The species found during the survey in the present study will be strong candidates for urban greening in Jingzhou due to their exploitable potential under natural conditions.

Jingzhou is a city that includes numerous ancient architectural structures and modern development. Attention must be paid to the degree and manner of management when managing green spaces in Jingzhou [40]. For areas such as Ji’nan which are in the process of urban development, SHPs should be protected and preserved as much as possible, existing in cities through various forms of patches. Due to the positive effect of edge effects, complex-shaped mosaics are beneficial for supporting more SHPs [49]. In the construction of building waste and idle land, SHPs with rapid growth can be used for temporary restoration [50,51]. Sufficient space for native plants should be reserved in newly constructed areas. Unlike Ji’nan, a highly urbanized area such as Shashi cannot accommodate the expansion of native plants by expanding the land area but can protect and preserve plants by transforming the original green space. The most important parameters to evaluate in the management of green space are not only ornamental, but also ecological safety and environmental benefits [27]. Selecting and using the original biological species that adapt to the city to replace those that are not adaptive and invasive is reasonable [46]. For urban areas with the potential for many plant living spaces, such as Gucheng, full use must be made of the fixed position of existing resources, such as the ancient city wall and the wetland resources of the moat belt. Moreover, native plants should be used to construct the wild landscape, especially three-dimensional greening [3], which contributes to the creation of a healthy ecological city and reflects the urban connotation.

It should be noted that in addition to ecological functions, SHPs are also bearers of traditional culture and folk customs [52]. Among the SHPs surveyed in Jingzhou, there are many plants that are closely related to people’s lives. *Artemisia argyi* and *A. caruifolia*, for example, were used as sacrifices in the Dragon Boat Festival to seek health and peace [53]. *Achillea millefolium* is a plant used for divination in ancient times, and also a medicinal plant [54]. There are many SHPs like this that are used and integrated into people’s lives and cultural heritage [55,56]. An ancient city that has gone through a long history and the vicissitudes of life is simple and unsophisticated under the growth of SHP communities. In ancient cities such as Jingzhou in China, which have many strong traditional cultural and spiritual beliefs and practices, the disappearance of these plants can also remove the cultural connotations they carry, potentially reducing the charm of these ancient cities.

## 4. Material and Methods

### 4.1. Study Area

The main urban area of Jingzhou is located on the Jianghan Plain, Hubei Province between (N: 30.210°, E: 112.100°) and (N: 30.450°, E: 112.410°) (Figure 1). It is an extension of the Shennongjia Forest District, an area of high biodiversity, and Dahong Mountain in Jingshan. Jingzhou is an important hub connecting north, south, east, and west in the hinterland of central China. It is an important transportation node located in the middle reaches of the Yangtze River and has been rated as one of the first historically and culturally distinguished cities in China. It is the capital with the longest history of the dynasty of Chu in the Spring and Autumn Period and the Warring States Period.

**Figure 8:**
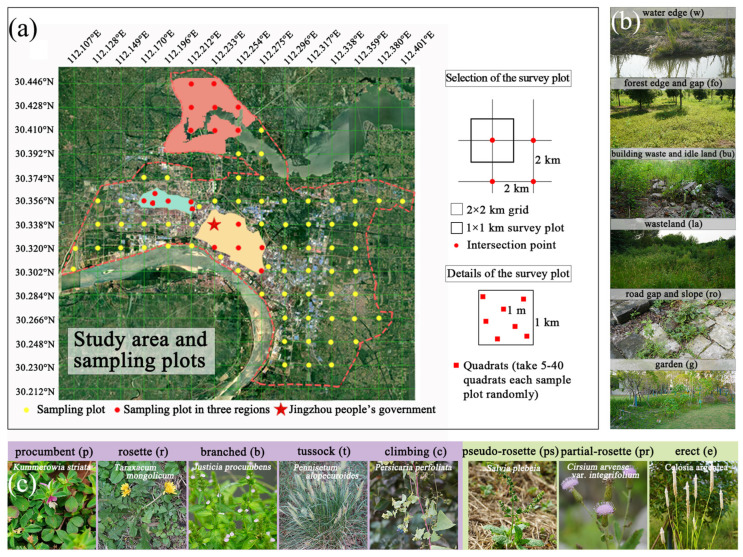
(**a**) Study area, sampling plots, and details of the survey plot. (**b**) The 6 colonizing habitat types. (**c**) The 8 studied plant growth forms and their typical species.

The geographical location of Jingzhou City creates a humid and mild environment suitable for the growth of a large number and diversity of native plants (Figure 8a). The city has a subtropical humid monsoon climate, with abundant rainfall, simultaneous rain and heat, an annual rainfall of 1000–1300 mm, and an annual average temperature of about 16 °C. May to June are the months with the highest rainfall, accounting for, on average, 51.4% of the annual rainfall.

The urban areas of Ji’nan (JN), Gucheng (GC), and Shashi (SS) were selected as the three main regions representative of current management modes in Jingzhou. The land use history of the three regions is different. Ji’nan is in the north of the city and is being developed as a cultural and tourism zone, and it is under construction. It is the site of the ancient Ji’nan city of Chu, adjacent to Changhu Wetland Park, and has rich water systems. Gucheng is located west of the main urban area of Jingzhou and is the official ship wharf of the ancient Chu State. The city wall buildings of Gucheng and their surrounding areas are in an ancient state. Shashi is a relatively developed and modern region in Jingzhou, developed from the old industrial zone, and has a high degree of urbanization.

### 4.2. Data Collection

This study investigated SHP species diversity in the entire Jingzhou main urban area through a field survey using a grid method. A 2 × 2 km grid was set with the Jingzhou people’s government as the origin (N: 30.338°, E: 112.233°), and a survey swath of 1 × 1 km was set with the grid crossing point as the center, with a total of 76 survey plots. Seven significant nodes were additionally supplemented as plots, resulting in a total of 83 plots (Supplementary Table S2); the central coordinates and the range of each sample plot were calculated.

For the three regions Ji’nan, Gucheng, and Shashi, the method of combining the sample method and inspection method was adopted. The information on the 19 sample plots of these three regions is listed in Supplementary Table S3. The random sampling method was adopted, 5–40 quadrats were sampled in each sample plot, and the quadrat was set as 1 × 1 m. The species name, quantity, viability, life form, growth form, and habitat of spontaneous herbaceous plants in the sample were recorded. Plant botanical nomenclature followed the system used in the Flora of China [57]. Due to the urbanization construction of the survey area, some areas were inaccessible for the survey, resulting in differences in the number of samples at the same plot between the spring and autumn and differences in quadrants and inspections between the spring and autumn. A total of 641 quadrats (124 in Ji’nan, 224 in Gucheng, 294 in Shashi) were investigated in the three regions in spring and autumn. The inspection method is adopted for the other 64 sample plots. For these sample plots, only the species name, life form, growth form, and habitat information of spontaneous herbaceous plants were investigated.

When conducting the field survey, the survey sample site was located according to GPS longitude and latitude coordinates for investigation. The survey cycle was divided into autumn and spring, from August to October 2021 and from March to May 2022, respectively.

### 4.3. Data Analysis

#### 4.3.1. The Composition of Families and Genera

Referring to the China Plant Science Data Center (https://www.plantplus.cn/cn (accessed on 29 August 2023)), we confirmed the scientific names of species, families, and genera, and we classified and counted all species of Jingzhou according to the APG Ⅳ classification system. Moreover, the plant species in the three regions were compared.

#### 4.3.2. Habitat Distribution

Referring to the habitat division methods of Chen et al. [58] and Li et al. [59], the main habitats were divided into six categories: water edge (w), forest edge and gap (fo), building waste and idle land (bu), wasteland (la), road gap and slope (ro), and garden (g) (Figure 8b). The SHP species in different habitats were counted and compared.

#### 4.3.3. Growth Form and Life Form Analysis

The growth forms of SHPs were divided into small growth forms and large growth forms; small growth forms included procumbent (p), rosette (r), branched (b), tussock (t), and climbing (c), and large growth forms included pseudo-rosette (ps), partial rosette (pr), and erect (e) (Figure 8c). The life forms were divided into annual (winter annual and summer annual) and perennial. The growth forms and life forms of plants were counted and compared.

#### 4.3.4. Species Diversity Analysis

We collected the spring and autumn quadrat data of the three regions and combined them for species diversity analysis. The Simpson dominance index (*D*), Shannon diversity index (*H*), and Pielou uniformity index (*J_SW_*) were used for the analysis of the alpha diversity index of the three regions [60]. The Jaccard similarity index (*J*) was used for the analysis of the beta diversity index [61]. When there was a significant difference between the groups, the Wilcoxon test was used for multiple comparisons.

Origin 2022 and the “ggplot 2” package in R (v. 4.3.1) were used for all data analysis and drawing [62].

## 5. Conclusions

SHPs play an important role in urban plant diversity. In the present study, we conducted a comprehensive survey of the diversity of SHPs in the main urban area of Jingzhou, paying particular attention to the growth forms and life forms of SHPs in different habitats. At the same time, we focused on the richness and diversity differences of SHPs in three typical regions. China has many cities with profound historical and cultural heritage, such as Jingzhou, and the conservation of spontaneous herbaceous plants in these cities is worthy of attention.

In the diverse habitats, there are 352 plant species belonging to 70 families and 236 genera in Jingzhou. Diverse habitats provide abundant living space for SHPs of different growth forms and life forms. Water edges with higher humidity do not significantly support more SHP growth forms and life forms, except for pseudo-rosette, partial-rosette, and perennial plants. In addition, both wasteland and road gaps and slopes support significantly more SHP growth forms, including erect, tussock, and others. Wasteland supported the vast majority of species, both growth forms and life forms. Different management levels have resulted in differences in the diversity of SHPs in Jingzhou. The Ji’nan region has had less interference than the Gucheng and Shashi regions and is rich in species. SHPs in urban areas are a conservation issue, and the concept of conservation and coordinated development of green areas should be implemented in new construction areas. The management of alien plants should be strengthened in restricted built-up areas, and native species with excellent performance in adapting to urbanization should be utilized. Consider that knowledge of the history of plants in the urban area is an additional aid to better understand which plants should be preserved. Our study provides decision-makers with new insights into the management of SHPs, especially those species that they think are weeds. Reasonable use and preservation of these plants in urban areas is key. It is strongly recommended that local SHPs be fully considered in the planning and management of green spaces, and urban landscape planning and ecological protection should be carried out according to local conditions.

## Data Availability

All the data supporting the findings of this study are included in this article.

## Supplementary Materials

The following supporting information can be downloaded at: https://www.mdpi.com/article/10.3390/plants12193353/s1, Table S1: List of spontaneous herbaceous plants in Jingzhou; Table S2: Setting of sample plots; Table S3: Sample plots information of three regions.
