# Supplementary material for: The Impact of Urbanization on the Distribution of Spontaneous Herbaceous Plants in an Ancient City: A Pilot Case Study in Jingzhou, China
Source: Plants (Basel). 2023 Sep 22;12(19):3353. doi: 10.3390/plants12193353 (PMC10574480; doi:10.3390/plants12193353)
Supplement: Supplementary file 1 [file plants-12-03353-s001.zip › plants-2611487-supplementary/Supplementary table0914.docx]

**Supplementary table 1** List of spontaneous herbaceous plants in Jingzhou

| **No.** | **Family name** | **Genus name** | **Scientific name** |
| --- | --- | --- | --- |
| 1 | Acanthaceae | Asystasia | *Asystasia gangetica* |
| 2 | Acanthaceae | Justicia | *Justicia procumbens* |
| 3 | Adoxaceae | Sambucus | *Sambucus javanica* |
| 4 | Alismataceae | Sagittaria | *Sagittaria trifolia*subsp.*Leucopetala* |
| 5 | Amaranthaceae | Alternanthera | *Alternanthera philoxeroides* |
| 6 | Amaranthaceae | Amaranthus | *Achyranthes bidentata* |
| 7 | Amaranthaceae | Amaranthus | *Amaranthus blitum* |
| 8 | Amaranthaceae | Amaranthus | *Amaranthus cruentus* |
| 9 | Amaranthaceae | Amaranthus | *Amaranthus hybridus* |
| 10 | Amaranthaceae | Amaranthus | *Amaranthus retroflexus* |
| 11 | Amaranthaceae | Celosia | *Celosia argentea* |
| 12 | Amaranthaceae | Chenopodium | *Chenopodium album* |
| 13 | Amaranthaceae | Chenopodium | *Chenopodium ficifolium* |
| 14 | Amaranthaceae | Chenopodium | *Chenopodium glaucum* |
| 15 | Amaranthaceae | Dysphania | *Dysphania ambrosioides* |
| 16 | Amaranthaceae | Dysphania | *Dysphania schraderiana* |
| 17 | Amaranthaceae | Kochia | *Kochia scoparia* |
| 18 | Amaranthaceae | Suaeda | *Suaeda salsa* |
| 19 | Apiaceae | Cnidium | *Cnidium monnieri* |
| 20 | Apiaceae | Ligusticum | *Ligusticum jeholense* |
| 21 | Apiaceae | Torilis | *Torilis scabra* |
| 22 | Apiaceae | Cicuta | *Cicuta virosa* |
| 23 | Apiaceae | Cyclospermum | *Cyclospermum leptophyllum* |
| 24 | Apiaceae | Oenanthe | *Oenanthe javanica* |
| 25 | Apocynaceae | Cynanchum | *Cynanchum auriculatum* |
| 26 | Apocynaceae | Metaplexis | *Metaplexis hemsleyana* |
| 27 | Apocynaceae | Metaplexis | *Metaplexis japonica* |
| 28 | Apocynaceae | Telosma | *Telosma cordata* |
| 29 | Apocynaceae | Trachelospermum | *Trachelospermum jasminoides* |
| 30 | Apocynaceae | Vinca | *Vinca major* |
| 31 | Apocynaceae | Vinca | *Vinca major 'Variegata'* |
| 32 | Araceae | Pinellia | *Pinellia pedatisecta* |
| 33 | Araceae | Pinellia | *Pinellia ternata* |
| 34 | Araliaceae | Hydrocotyle | *Hydrocotyle sibthorpioides* |
| 35 | Araliaceae | Hydrocotyle | *Hydrocotyle sibthorpioides*var.*batrachium* |
| 36 | Asparagaceae | Asparagus | *Asparagus cochinchinensis* |
| 37 | Asparagaceae | Ophiopogon | *Ophiopogon japonicus* |
| 38 | Asphodelaceae | Hemerocallis | *Hemerocallis fulva* |
| 39 | Asteraceae | Adenostemma | *Adenostemma lavenia* |
| 40 | Asteraceae | Artemisia | *Artemisia argyi* |
| 41 | Asteraceae | Artemisia | *Artemisia caruifolia* |
| 42 | Asteraceae | Artemisia | *Artemisia selengensis* |
| 43 | Asteraceae | Aster | *Aster baccharoides* |
| 44 | Asteraceae | Aster | *Aster indicus* |
| 45 | Asteraceae | Aster | *Aster lautureanus* |
| 46 | Asteraceae | Bidens | *Bidens pilosa* |
| 47 | Asteraceae | Carpesium | *Carpesium abrotanoides* |
| 48 | Asteraceae | Centipeda | *Centipeda minima* |
| 49 | Asteraceae | Chrysanthemum | *Chrysanthemum indicum* |
| 50 | Asteraceae | Cirsium | *Cirsium arvense*var.*integrifolium* |
| 51 | Asteraceae | Cirsium | *Cirsium japonicum* |
| 52 | Asteraceae | Cirsium | *Cirsium vlassovianum* |
| 53 | Asteraceae | Eclipta | *Eclipta prostrata* |
| 54 | Asteraceae | Emilia | *Emilia prenanthoidea* |
| 55 | Asteraceae | Erigeron | *Erigeron annuus* |
| 56 | Asteraceae | Erigeron | *Erigeron bonariensis* |
| 57 | Asteraceae | Erigeron | *Erigeron canadensis* |
| 58 | Asteraceae | Galinsoga | *Galinsoga parviflora* |
| 59 | Asteraceae | Gynura | *Gynura divaricata* |
| 60 | Asteraceae | Helianthus | *Helianthus tuberosus* |
| 61 | Asteraceae | Hemisteptia | *Hemisteptia lyrata* |
| 62 | Asteraceae | Inula | *Inula japonica* |
| 63 | Asteraceae | Ixeris | *Ixeris chinensis* |
| 64 | Asteraceae | Ixeris | *Ixeris polycephala* |
| 65 | Asteraceae | Lactuca | *Lactuca indica* |
| 66 | Asteraceae | Lactuca | *Lactuca sibirica* |
| 67 | Asteraceae | Praxelis | *Praxelis clematidea* |
| 68 | Asteraceae | Pseudognaphalium | *Pseudognaphalium affine* |
| 69 | Asteraceae | Senecio | *Senecio scandens* |
| 70 | Asteraceae | Sonchus | *Sonchus asper* |
| 71 | Asteraceae | Sonchus | *Sonchus oleraceus* |
| 72 | Asteraceae | Sonchus | *Sonchus wightianus* |
| 73 | Asteraceae | Symphyotrichum | *Symphyotrichum subulatum* |
| 74 | Asteraceae | Taraxacum | *Taraxacum mongolicum* |
| 75 | Asteraceae | Xanthium | *Xanthium strumarium* |
| 76 | Asteraceae | Youngia | *Youngia heterophylla* |
| 77 | Asteraceae | Ageratum | *Ageratum conyzoides* |
| 78 | Asteraceae | Artemisia | *Artemisia annua* |
| 79 | Asteraceae | Carduus | *Carduus nutans* |
| 80 | Asteraceae | Lactuca | *Lactuca serriola* |
| 81 | Asteraceae | Solidago | *Solidago canadensis* |
| 82 | Asteraceae | Youngia | *Youngia japonica* |
| 83 | Basellaceae | Basella | *Basella alba* |
| 84 | Boraginaceae | Bothriospermum | *Bothriospermum zeylanicum* |
| 85 | Boraginaceae | Thyrocarpus | *Thyrocarpus glochidiatus* |
| 86 | Boraginaceae | Bothriospermum | *Bothriospermum chinense* |
| 87 | Boraginaceae | Trigonotis | *Trigonotis peduncularis* |
| 88 | Brassicaceae | Brassica | *Brassica juncea* |
| 89 | Brassicaceae | Capsella | *Capsella bursa-pastoris* |
| 90 | Brassicaceae | Cardamine | *Cardamine hirsuta* |
| 91 | Brassicaceae | Lepidium | *Lepidium apetalum* |
| 92 | Brassicaceae | Lepidium | *Lepidium didymum* |
| 93 | Brassicaceae | Orychophragmus | *Orychophragmus violaceus* |
| 94 | Brassicaceae | Rorippa | *Rorippa cantoniensis* |
| 95 | Brassicaceae | Rorippa | *Rorippa indica* |
| 96 | Campanulaceae | Lobelia | *Lobelia chinensis* |
| 97 | Cannabaceae | Humulus | *Humulus scandens* |
| 98 | Caprifoliaceae | Lonicera | *Lonicera japonica* |
| 99 | Caryophyllaceae | Arenaria | *Arenaria serpyllifolia* |
| 100 | Caryophyllaceae | Sagina | *Sagina japonica* |
| 101 | Caryophyllaceae | Cerastium | *Cerastium fontanum* |
| 102 | Caryophyllaceae | Myosoton | *Myosoton aquaticum* |
| 103 | Caryophyllaceae | Stellaria | *Stellaria media* |
| 104 | Celastraceae | Celastrus | *Celastrus orbiculatus* |
| 105 | Celastraceae | Euonymus | *Euonymus fortunei* |
| 106 | Cleomaceae | Arivela | *Arivela viscosa* |
| 107 | Commelinaceae | Commelina | *Commelina communis* |
| 108 | Convolvulaceae | Calystegia | *Calystegia hederacea* |
| 109 | Convolvulaceae | Cuscuta | *Cuscuta chinensis* |
| 110 | Convolvulaceae | Dichondra | *Dichondra micrantha* |
| 111 | Convolvulaceae | Ipomoea | *Ipomoea nil* |
| 112 | Convolvulaceae | Ipomoea | *Ipomoea obscura* |
| 113 | Convolvulaceae | Ipomoea | *Ipomoea quamoclit* |
| 114 | Convolvulaceae | Ipomoea | *Ipomoea triloba* |
| 115 | Crassulaceae | Sedum | *Sedum bulbiferum* |
| 116 | Crassulaceae | Sedum | *Sedum hispanicum* |
| 117 | Crassulaceae | Sedum | *Sedum stellariifolium* |
| 118 | Crassulaceae | Sedum | *Sedum emarginatum* |
| 119 | Crassulaceae | Sedum | *Sedum sarmentosum* |
| 120 | Cucurbitaceae | Actinostemma | *Actinostemma tenerum* |
| 121 | Cucurbitaceae | Thladiantha | *Thladiantha dubia* |
| 122 | Cucurbitaceae | Trichosanthes | *Trichosanthes kirilowii* |
| 123 | Cucurbitaceae | Zehneria | *Zehneria japonica* |
| 124 | Cyperaceae | Cyperus | *Cyperus compressus* |
| 125 | Cyperaceae | Cyperus | *Cyperus difformis* |
| 126 | Cyperaceae | Cyperus | *Cyperus glomeratus* |
| 127 | Cyperaceae | Cyperus | *Cyperus imbricatus* |
| 128 | Cyperaceae | Cyperus | *Cyperus iria* |
| 129 | Cyperaceae | Cyperus | *Cyperus michelianus* |
| 130 | Cyperaceae | Cyperus | *Cyperus microiria* |
| 131 | Cyperaceae | Cyperus | *Cyperus papyrus* |
| 132 | Cyperaceae | Cyperus | *Cyperus rotundus* |
| 133 | Cyperaceae | Fimbristylis | *Fimbristylis littoralis* |
| 134 | Cyperaceae | Fimbristylis | *Fimbristylis dichotoma* |
| 135 | Cyperaceae | Kyllinga | *Kyllinga brevifolia* |
| 136 | Cyperaceae | Pycreus | *Pycreus flavidus* |
| 137 | Cyperaceae | Pycreus | *Pycreus sanguinolentus* |
| 138 | Cyperaceae | Schoenoplectus | *Schoenoplectus tabernaemontani* |
| 139 | Cyperaceae | Schoenoplectus | *Schoenoplectus triqueter* |
| 140 | Cyperaceae | Carex | *Carex heterostachya* |
| 141 | Cyperaceae | Carex | *Carex rhynchophysa* |
| 142 | Equisetaceae | Equisetum | *Equisetum ramosissimum* |
| 143 | Equisetaceae | Equisetum | *Equisetum ramosissimum* |
| 144 | Euphorbiaceae | Euphorbia | *Euphorbia helioscopia* |
| 145 | Euphorbiaceae | Euphorbia | *Euphorbia humifusa* |
| 146 | Euphorbiaceae | Euphorbia | *Euphorbia hypericifolia* |
| 147 | Euphorbiaceae | Euphorbia | *Euphorbia thymifolia* |
| 148 | Euphorbiaceae | Acalypha | *Acalypha australis* |
| 149 | Euphorbiaceae | Euphorbia | *Euphorbia hirta* |
| 150 | Euphorbiaceae | Euphorbia | *Euphorbia maculata* |
| 151 | Euphorbiaceae | Ricinus | *Ricinus communis* |
| 152 | Fabaceae | Aeschynomene | *Aeschynomene indica* |
| 153 | Fabaceae | Kummerowia | *Kummerowia striata* |
| 154 | Fabaceae | Medicago | *Medicago polymorpha* |
| 155 | Fabaceae | Rhynchosia | *Rhynchosia volubilis* |
| 156 | Fabaceae | Senna | *Senna tora* |
| 157 | Fabaceae | Vicia | *Vicia sepium* |
| 158 | Fabaceae | Vigna | *Vigna radiata* |
| 159 | Fabaceae | Astragalus | *Astragalus sinicus* |
| 160 | Fabaceae | Glycine | *Glycine soja* |
| 161 | Fabaceae | Medicago | *Medicago sativa* |
| 162 | Fabaceae | Melilotus | *Melilotus officinalis* |
| 163 | Fabaceae | Pueraria | *Pueraria montana* |
| 164 | Fabaceae | Trifolium | *Trifolium repens* |
| 165 | Fabaceae | Vicia | *Vicia cracca* |
| 166 | Fabaceae | Vicia | *Vicia hirsuta* |
| 167 | Fabaceae | Vicia | *Vicia sativa* |
| 168 | Geraniaceae | Geranium | *Geranium sibiricum* |
| 169 | Geraniaceae | Geranium | *Geranium wilfordii* |
| 170 | Haloragaceae | Myriophyllum | *Myriophyllum verticillatum* |
| 171 | Hydrocharitaceae | Hydrocharis | *Hydrocharis dubia* |
| 172 | Juncaceae | Juncus | *Juncus effusus* |
| 173 | Lamiaceae | Ajuga | *Ajuga multiflora* |
| 174 | Lamiaceae | Anisomeles | *Anisomeles indica* |
| 175 | Lamiaceae | Clinopodium | *Clinopodium chinense* |
| 176 | Lamiaceae | Clinopodium | *Clinopodium megalanthum* |
| 177 | Lamiaceae | Isodon | *Isodon excisus* |
| 178 | Lamiaceae | Lamium | *Lamium barbatum* |
| 179 | Lamiaceae | Leonurus | *Leonurus japonicus* |
| 180 | Lamiaceae | Mosla | *Mosla dianthera* |
| 181 | Lamiaceae | Ocimum | *Ocimum basilicum* |
| 182 | Lamiaceae | Perilla | *Perilla frutescens* |
| 183 | Lamiaceae | Perilla | *Perilla frutescens*var.*purpurascens* |
| 184 | Lamiaceae | Salvia | *Salvia plebeia* |
| 185 | Lamiaceae | Mentha | *Mentha canadensis* |
| 186 | Lamiaceae | Prunella | *Prunella vulgaris* |
| 187 | Lamiaceae | Stachys | *Stachys oblongifolia* |
| 188 | Lardizabalaceae | Akebia | *Akebia quinata* |
| 189 | Lardizabalaceae | Akebia | *Akebia trifoliata* |
| 190 | Linderniaceae | Lindernia | *Lindernia anagallis* |
| 191 | Linderniaceae | Lindernia | *Lindernia antipoda* |
| 192 | Linderniaceae | Lindernia | *Lindernia crustacea* |
| 193 | Linderniaceae | Lindernia | *Lindernia nummulariifolia* |
| 194 | Linderniaceae | Lindernia | *Lindernia procumbens* |
| 195 | Lygodiaceae | Lygodium | *Lygodium japonicum* |
| 196 | Lythraceae | Ammannia | *Ammannia baccifera* |
| 197 | Lythraceae | Lythrum | *Lythrum salicaria* |
| 198 | Malvaceae | Abelmoschus | *Abelmoschus esculentus* |
| 199 | Malvaceae | Abutilon | *Abutilon theophrasti* |
| 200 | Malvaceae | Grewia | *Grewia biloba* |
| 201 | Malvaceae | Melochia | *Melochia corchorifolia* |
| 202 | Malvaceae | Corchorus | *Corchorus aestuans* |
| 203 | Malvaceae Juss. | Sida szechuensis Matsuda | *Sida cordifolia* |
| 204 | Marsileaceae | Marsilea | *Marsilea quadrifolia* |
| 205 | Mazaceae | Mazus | *Mazus pumilus* |
| 206 | Menispermaceae | Cocculus | *Cocculus orbiculatus* |
| 207 | Menispermaceae | Pericampylus | *Pericampylus glaucus* |
| 208 | Molluginaceae | Trigastrotheca | *Trigastrotheca stricta* |
| 209 | Nyctaginaceae | Mirabilis | *Mirabilis jalapa* |
| 210 | Onagraceae | Oenothera | *Oenothera biennis* |
| 211 | Onagraceae | Ludwigia | *Ludwigia prostrata* |
| 212 | Oxalidaceae | Oxalis | *Oxalis corniculata* |
| 213 | Oxalidaceae | Oxalis | *Oxalis corymbosa* |
| 214 | Oxalidaceae | Oxalis | *Oxalis pes-caprae* |
| 215 | Papaveraceae | Corydalis | *Corydalis decumbens* |
| 216 | Papaveraceae | Corydalis | *Corydalis edulis* |
| 217 | Phyllanthaceae | Phyllanthus | *Phyllanthus urinaria* |
| 218 | Phyllanthaceae | Phyllanthus | *Phyllanthus ussuriensis* |
| 219 | Phytolaccaceae | Phytolacca | *Phytolacca acinosa* |
| 220 | Phytolaccaceae | Phytolacca | *Phytolacca americana* |
| 221 | Plantaginaceae | Kickxia | *Kickxia elatine* |
| 222 | Plantaginaceae | Plantago | *Plantago asiatica* |
| 223 | Plantaginaceae | Veronica | *Veronica arvensis* |
| 224 | Plantaginaceae | Veronica | *Veronica polita* |
| 225 | Plantaginaceae | Veronica | *Veronica undulata* |
| 226 | Plantaginaceae | Veronica | *Veronica persica* |
| 227 | Poaceae | Alopecurus | *Alopecurus aequalis* |
| 228 | Poaceae | Arthraxon | *Arthraxon hispidus* |
| 229 | Poaceae | Arundo | *Arundo donax* |
| 230 | Poaceae | Beckmannia | *Beckmannia syzigachne* |
| 231 | Poaceae | Bromus | *Bromus japonicus* |
| 232 | Poaceae | Chrysopogon | *Chrysopogon aciculatus* |
| 233 | Poaceae | Digitaria | *Digitaria sanguinalis* |
| 234 | Poaceae | Echinochloa | *Echinochloa crus-galli* |
| 235 | Poaceae | Echinochloa | *Echinochloa crus-galli*var.*austrojaponensis* |
| 236 | Poaceae | Eragrostis | *Eragrostis ferruginea* |
| 237 | Poaceae | Eremochloa | *Eremochloa ciliaris* |
| 238 | Poaceae | Leersia | *Leersia hexandra* |
| 239 | Poaceae | Lolium | *Lolium perenne* |
| 240 | Poaceae | Pennisetum | *Pennisetum alopecuroides* |
| 241 | Poaceae | Phragmites | *Phragmites australis* |
| 242 | Poaceae | Poa | *Poa acroleuca* |
| 243 | Poaceae | Polypogon | *Polypogon fugax* |
| 244 | Poaceae | Setaria | *Setaria pumila* |
| 245 | Poaceae | Triticum | *Triticum aestivum* |
| 246 | Poaceae | Avena | *Avena fatua* |
| 247 | Poaceae | Coix | *Coix lacryma-jobi* var.*ma-yuen* |
| 248 | Poaceae | Cynodon | *Cynodon dactylon* |
| 249 | Poaceae | Digitaria | *Digitaria violascens* |
| 250 | Poaceae | Echinochloa | *Echinochloa caudata* |
| 251 | Poaceae | Echinochloa | *Echinochloa colona* |
| 252 | Poaceae | Eleusine | *Eleusine indica* |
| 253 | Poaceae | Elymus | *Elymus kamoji* |
| 254 | Poaceae | Eragrostis | *Eragrostis minor* |
| 255 | Poaceae | Eragrostis | *Eragrostis pilosa* |
| 256 | Poaceae | Eriochloa | *Eriochloa villosa* |
| 257 | Poaceae | Festuca | *Festuca elata* |
| 258 | Poaceae | Imperata | *Imperata cylindrica* |
| 259 | Poaceae | Leptochloa | *Leptochloa chinensis* |
| 260 | Poaceae | Leptochloa | *Leptochloa panicea* |
| 261 | Poaceae | Miscanthus | *Miscanthus sacchariflorus* |
| 262 | Poaceae | Oplismenus | *Oplismenus compositus* |
| 263 | Poaceae | Panicum | *Panicum bisulcatum* |
| 264 | Poaceae | Panicum | *Panicum miliaceum* |
| 265 | Poaceae | Panicum | *Panicum sumatrense* |
| 266 | Poaceae | Paspalum | *Paspalum distichum* |
| 267 | Poaceae | Paspalum | *Paspalum scrobiculatum*var. *orbiculare* |
| 268 | Poaceae | Paspalum | *Paspalum thunbergii* |
| 269 | Poaceae | Poa | *Poa annua* |
| 270 | Poaceae | Poa | *Poa nemoralis* |
| 271 | Poaceae | Setaria | *Setaria faberi* |
| 272 | Poaceae | Setaria | *Setaria viridis* |
| 273 | Poaceae | Sorghum | *Sorghum bicolor* |
| 274 | Poaceae | Sporobolus | *Sporobolus fertilis* |
| 275 | Poaceae | Zizania | *Zizania latifolia* |
| 276 | Polemoniaceae | Phlox | *Phlox subulata* |
| 277 | Polygonaceae | Rumex | *Rumex crispus* |
| 278 | Polygonaceae | Rumex | *Rumex japonicus* |
| 279 | Polygonaceae | Fagopyrum | *Fagopyrum dibotrys* |
| 280 | Polygonaceae | Fallopia | *Fallopia multiflora* |
| 281 | Polygonaceae | Polygonum | *Polygonum aviculare* |
| 282 | Polygonaceae | Polygonum | *Polygonum barbatum* |
| 283 | Polygonaceae | Polygonum | *Polygonum hydropiper* |
| 284 | Polygonaceae | Polygonum | *Polygonum japonicum* |
| 285 | Polygonaceae | Polygonum | *Polygonum jucundum* |
| 286 | Polygonaceae | Polygonum | *Polygonum lapathifolium* |
| 287 | Polygonaceae | Polygonum | *Polygonum lapathifolium*var.*salicifolium* |
| 288 | Polygonaceae | Polygonum | *Polygonum orientale* |
| 289 | Polygonaceae | Polygonum | *Polygonum perfoliatum* |
| 290 | Polygonaceae | Polygonum | *Polygonum posumbu* |
| 291 | Polygonaceae | Polygonum | *Polygonum senticosum* |
| 292 | Polygonaceae | Polygonum | *Polygonum viscosum* |
| 293 | Polygonaceae | Rumex | *Rumex acetosa* |
| 294 | Polygonaceae | Rumex | *Rumex dentatus* |
| 295 | Polygonaceae | Rumex | *Rumex trisetifer Stokes* |
| 296 | Pontederiaceae | Eichhornia | *Eichhornia crassipes* |
| 297 | Portulacaceae | Portulaca | *Portulaca oleracea* |
| 298 | Primulaceae | Lysimachia | *Lysimachia grammica* |
| 299 | Primulaceae | Lysimachia | *Lysimachiafortunei* |
| 300 | Primulaceae | Lysimachia | *Lysimachia candida* |
| 301 | Primulaceae | Lysimachia | *Lysimachia christiniae* |
| 302 | Primulaceae | Lysimachia | *Lysimachia heterogenea* |
| 303 | Pteridaceae | Pteris | *Pteris multifida* |
| 304 | Ranunculaceae | Clematis | *Clematis florida* |
| 305 | Ranunculaceae | Ranunculus | *Ranunculus japonicus* |
| 306 | Ranunculaceae | Ranunculus | *Ranunculus sceleratus* |
| 307 | Ranunculaceae | Ranunculus | *Ranunculus sieboldii* |
| 308 | Ranunculaceae | Semiaquilegia | *Semiaquilegia adoxoides* |
| 309 | Ranunculaceae | Ranunculus | *Ranunculus chinensis* |
| 310 | Rosaceae | Potentilla | *Potentilla supina* |
| 311 | Rosaceae | Rubus | *Rubus parvifolius* |
| 312 | Rosaceae | Rubus | *Rubus innominatus* |
| 313 | Rubiaceae | Duchesnea | *Duchesnea indica* |
| 314 | Rubiaceae | Fragaria | *Fragaria vesca* |
| 315 | Rubiaceae | Galium | *Galium bungei* |
| 316 | Rubiaceae | Rosa | *Rosa multiflora* |
| 317 | Rubiaceae | Rubia | *Rubia cordifolia* |
| 318 | Rubiaceae | Galium | *Galium odoratum* |
| 319 | Rubiaceae | Galium | *Galium spurium* |
| 320 | Rubiaceae | Hedyotis | *Hedyotis diffusa* |
| 321 | Rubiaceae | Paederia | *Paederia foetida* |
| 322 | Rutaceae | Zanthoxylum | *Zanthoxylum nitidum* |
| 323 | Sapindaceae | Cardiospermum | *Cardiospermum halicacabum* |
| 324 | Saururaceae | Houttuynia | *Houttuynia cordata* |
| 325 | Solanaceae | Nicandra | *Nicandra physalodes* |
| 326 | Solanaceae | Physalis | *Physalis angulata* |
| 327 | Solanaceae | Solanum | *Solanum lyratum* |
| 328 | Solanaceae | Solanum | *Solanum nigrum* |
| 329 | Solanaceae | Lycium | *Lycium chinense* |
| 330 | Talinaceae | Talinum | *Talinum paniculatum* |
| 331 | Thelypteridaceae | Cyclosorus | *Cyclosorus acuminatus* |
| 332 | Typhaceae | Typha | *Typha minima* |
| 333 | Typhaceae | Typha | *Typha orientalis* |
| 334 | Urticaceae | Boehmeria | *Boehmeria nivea* |
| 335 | Urticaceae | Debregeasia | *Debregeasia orientalis* |
| 336 | Urticaceae | Nanocnide | *Nanocnide lobata* |
| 337 | Urticaceae | Pilea | *Pilea microphylla* |
| 338 | Urticaceae | Pilea | *Pilea notata* |
| 339 | Urticaceae | Pouzolzia | *Pouzolzia zeylanica* |
| 340 | Urticaceae | Pouzolzia | *Pouzolzia zeylanica*var. *microphylla* |
| 341 | Verbenaceae | Phyla | *Phyla nodiflora* |
| 342 | Verbenaceae | Verbena | *Verbena bonariensis* |
| 343 | Verbenaceae | Verbena | *Verbena officinalis* |
| 344 | Violaceae | Viola | *Viola betonicifolia* |
| 345 | Violaceae | Viola | *Viola philippica* |
| 346 | Violaceae | Viola | *Viola yunnanfuensis* |
| 347 | Vitaceae | Ampelopsis | *Ampelopsis delavayana* |
| 348 | Vitaceae | Ampelopsis | *Ampelopsis glandulosa* |
| 349 | Vitaceae | Parthenocissus | *Parthenocissus semicordata* |
| 350 | Vitaceae | Vitis | *Vitis bryoniifolia* |
| 351 | Vitaceae | Cayratia | *Cayratia japonica* |
| 352 | Vitaceae | Parthenocissus | *Parthenocissus tricuspidata* |

**Supplementary table 2** Setting of sample plots

| **Plot No.** | **Longitude and latitude** |
| --- | --- |
|  |  |
| 1 | N: 30.446 °, E: 112.212 ° |
| 2 | N: 30.446 °, E: 112.233 ° |
| 3 | N: 30.428 °, E: 112.212 ° |
| 5 | N: 30.428 °, E: 112.254 ° |
| 6 | N: 30.410 °, E: 112.212 ° |
| 7 | N: 30.410 °, E: 112.233 ° |
| 8 | N: 30.410 °, E: 112.254 ° |
| 9 | N: 30.410 °, E: 112.275 ° |
| 10 | N: 30.392 °, E: 112.254 ° |
| 11 | N: 30.392 °, E: 112.275 ° |
| 12 | N: 30.374 °, E: 112.170 ° |
| 13 | N: 30.374 °, E: 112.196 ° |
| 14 | N: 30.374 °, E: 112.254 ° |
| 15 | N: 30.374 °, E: 112.275 ° |
| 16 | N: 30.356 °, E: 112.128 ° |
| 17 | N: 30.356 °, E: 112.149 ° |
| 18 | N: 30.356 °, E: 112.170 ° |
| 19 | N: 30.356 °, E: 112.196 ° |
| 20 | N: 30.356 °, E: 112.212 ° |
| 21 | N: 30.356 °, E: 112.233 ° |
| 22 | N: 30.356 °, E: 112.254 ° |
| 23 | N: 30.356 °, E: 112.275 ° |
| 24 | N: 30.356 °, E: 112.296 ° |
| 25 | N: 30.356 °, E: 112.317 ° |
| 26 | N: 30.356 °, E: 112.338 ° |
| 27 | N: 30.356 °, E: 112.359 ° |
| 28 | N: 30.356 °, E: 112.380 ° |
| 29 | N: 30.356 °, E: 112.401 ° |
| 30 | N: 30.338 °, E: 112.128 ° |
| 31 | N: 30.338 °, E: 112.149 ° |
| 32 | N: 30.338 °, E: 112.170 ° |
| 33 | N: 30.338 °, E: 112.191 ° |
| 34 | N: 30.338 °, E: 112.212 ° |
| 35 | N: 30.338 °, E: 112.233 ° |
| 36 | N: 30.338 °, E: 112.254 ° |
| 37 | N: 30.338 °, E: 112.275 ° |
| 38 | N: 30.338 °, E: 112.296 ° |
| 39 | N: 30.338 °, E: 112.317 ° |
| 40 | N: 30.338 °, E: 112.338 ° |
| 41 | N: 30.338 °, E: 112.359 ° |
| 42 | N: 30.320 °, E: 112.107 ° |
| 43 | N: 30.320 °, E: 112.128 ° |
| 44 | N: 30.320 °, E: 112.149 ° |
| 45 | N: 30.320 °, E: 112.170 ° |
| 46 | N: 30.320 °, E: 112.233 ° |
| 47 | N: 30.320 °, E: 112.254 ° |
| 48 | N: 30.320 °, E: 112.275 ° |
| 49 | N: 30.320 °, E: 112.296 ° |
| 50 | N: 30.320 °, E: 112.317 ° |
| 51 | N: 30.320 °, E: 112.338 ° |
| 52 | N: 30.320 °, E: 112.359 ° |
| 53 | N: 30.302 °, E: 112.107 ° |
| 54 | N: 30.302 °, E: 112.254 ° |
| 55 | N: 30.302 °, E: 112.275 ° |
| 56 | N: 30.302 °, E: 112.296 ° |
| 57 | N: 30.302 °, E: 112.317 ° |
| 58 | N: 30.302 °, E: 112.338 ° |
| 59 | N: 30.302 °, E: 112.359 ° |
| 60 | N: 30.284 °, E: 112.275 ° |
| 61 | N: 30.284 °, E: 112.296 ° |
| 62 | N: 30.284 °, E: 112.317 ° |
| 63 | N: 30.284 °, E: 112.338 ° |
| 64 | N: 30.284 °, E: 112.359 ° |
| 65 | N: 30.266 °, E: 112.296 ° |
| 66 | N: 30.266 °, E: 112.317 ° |
| 67 | N: 30.266 °, E: 112.338 ° |
| 68 | N: 30.266 °, E: 112.359 ° |
| 69 | N: 30.266 °, E: 112.380 ° |
| 70 | N: 30.248 °, E: 112.296 ° |
| 71 | N: 30.248 °, E: 112.317 ° |
| 72 | N: 30.248 °, E: 112.338 ° |
| 73 | N: 30.248 °, E: 112.359 ° |
| 74 | N: 30.230 °, E: 112.296 ° |
| 75 | N: 30.230 °, E: 112.317 ° |
| 76 | N: 30.230 °, E: 112.338 ° |
| 80 | N: 30.320 °, E: 112,191 ° |
| 81 | N: 30.320 °, E: 112.212 ° |
| 82 | N: 30.352 °, E: 112.190 ° |
| 83 | N: 30.347 °, E: 112.218 ° |
| 84 | N: 30.360 °, E: 112.186 ° |
| 85 | N: 30.357 °, E: 112.198 ° |
| 86 | N: 30.310 °, E: 112.266 ° |
| 87 | N: 30.354 °, E: 112.138 ° |

**Supplementary table 3** Sample plots information of three regions

| **Regions** | **Plot No.** | **Longitude and latitude** | **Survey methods** | **Number of quadrats** | |
| --- | --- | --- | --- | --- | --- |
|  |  |  |  | **Spring** | **Autumn** |
| JN | 1 | N: 30.446 °, E: 112.212 ° | Quadrats and inspection | 24 | - |
| JN | 2 | N: 30.446 °, E: 112.233 ° | Inspection | - | - |
| JN | 3 | N: 30.428 °, E: 112.212 ° | Quadrats and inspection | 19 | 26 |
| JN | 5 | N: 30.428 °, E: 112.254 ° | Quadrats | 18 | 37 |
| JN | 6 | N: 30.410 °, E: 112.212 ° | Inspection | - | - |
| JN | 7 | N: 30.410 °, E: 112.233 ° | Inspection | - | - |
| JN | 8 | N: 30.410 °, E: 112.254 ° | Inspection | - | - |
| GC | 18 | N: 30.356 °, E: 112.170 ° | Quadrats | 13 | 38 |
| GC | 19 | N: 30.356 °, E: 112.196 ° | Quadrats | 14 | 16 |
| GC | 20 | N: 30.356 °, E: 112.212 ° | Quadrats | 31 | 52 |
| GC | 84 | N: 30.360 °, E: 112.186 ° | Quadrats | 14 | 8 |
| GC | 85 | N: 30.357 °, E: 112.198 ° | Quadrats and inspection | 16 | - |
| GC | 87 | N: 30.354 °, E: 112.138 ° | Quadrats | 11 | 11 |
| SS | 35 | N: 30.338 °, E: 112.233 ° | Quadrats | 29 | 30 |
| SS | 36 | N: 30.338 °, E: 112.254 ° | Quadrats | 18 | 21 |
| SS | 46 | N: 30.320 °, E: 112.233 ° | Quadrats | 22 | 33 |
| SS | 47 | N: 30.320 °, E: 112.254 ° | Quadrats | 29 | 14 |
| SS | 48 | N: 30.320 °, E: 112.275 ° | Quadrats | 20 | 35 |
| SS | 55 | N: 30.302 °, E: 112.275 ° | Quadrats | 10 | 32 |
